# Gynecological Diagnosis

**Published:** 1910-12

**Authors:** 


**Gynecological Diagnosis.** By **Walter L. Burrage,** A.M., M.D. (Harv.), Clinical Instructor in Gynecology in Harvard University. Octavo, pp. 672. With 207 illustrations. New York and London: D. Appleton & Co. 1910. (Cloth, $6.00.)

We need to learn more about diagnosis. An attempt has been made in this volume to keep in the background the rare diseases that are of interest only to the specialist, and to give prominence to the common affections usually met by the general practitioner and which form so large a part of his practice. All general constitutional diseases have a bearing both as causative agents and aggravating influences on pelvic diseases. It should never be forgotten that the whole is greater than any one part, and that in gynecological affections it is the sick woman we are to treat. Burrage has scanned the literature old and recent and all ideas of value are incorporated in his book. Special features are an explanation of the anatomy and mechanism of malposition of the uterus. The chapter on the menopause sheds light on a most important but little understood period of woman's life.

The chapter on the diagnosis of the diseases of the ovaries is very full, and the differential diagnosis is treated at length. A helpful feature is an alphabetical index of illustrations of which there are two hundred and fifteen, and every chapter is headed by a summary of its contents with page reference. J. A. R.

